# Travel-Related Typhoid Fever: Narrative Review of the Scientific Literature

**DOI:** 10.3390/ijerph17020615

**Published:** 2020-01-18

**Authors:** Narcisa Muresu, Giovanni Sotgiu, Bianca Maria Are, Andrea Cossu, Clementina Cocuzza, Marianna Martinelli, Sergio Babudieri, Riccardo Are, Marco Dettori, Antonio Azara, Laura Saderi, Andrea Piana

**Affiliations:** 1 Department of Biomedical Sciences, University of Sassari, 07100 Sassari, Italy; narcisamuresu@outlook.com; 2 Department of Medical, Surgical and Experimental Sciences, University of Sassari, 07100 Sassari, Italy; andreacossu@uniss.it (A.C.); madettori@uniss.it (M.D.); azara@uniss.it (A.A.); lsaderi@uniss.it (L.S.); piana@uniss.it (A.P.); 3 Hygiene and Preventive Medicine Unit, AOU Sassari, 07100 Sassari, Italy; arebm@uniss.it; 4 Department of Medicine and Surgery, University of Milano-Bicocca, Via Cadore 48, 20900 Monza, Italy; clementina.cocuzza@unimib.it (C.C.); marianna.martinelli@unimib.it (M.M.); 5 Infectious Diseases Department, AOU Sassari, University of Sassari, 07100 Sassari, Italy; babuder@uniss.it (S.B.); riccardo.are@aousassari.it (R.A.)

**Keywords:** salmonella enterica, typhoid and paratyphoid fever, travelers

## Abstract

Enteric fever is a foodborne infectious disease caused by Salmonella enterica serotypes *Typhi* and *Paratyphi* A, B and C. The high incidence in low income countries can increase the risk of disease in travelers coming from high income countries. Pre-travel health advice on hygiene and sanitation practices and vaccines can significantly reduce the risk of acquiring infections. Although the majority of the cases are self-limiting, life-threatening complications can occur. Delayed diagnosis and cases of infections caused by multi-drug resistant strains can complicate the clinical management and affect the prognosis. More international efforts are needed to reduce the burden of disease in low income countries, indirectly reducing the risk of travelers in endemic settings. Surveillance activities can help monitor the epidemiology of cases caused by drug-susceptible and resistant strains.

## 1. Introduction

In the context of a globalization process, travels can pose a threat to the health of millions of persons worldwide. Outbreaks and epidemic episodes of transmissible diseases (e.g., Ebola, Zika, Middle East respiratory syndrome, etc.), potentially associated with travel of population groups, have raised the attention of supranational and national governments based on their mortality, morbidity, and impact on the sustainability of country and regional healthcare systems. Migration waves and business or holiday travels might be the epidemiological driver of several infectious diseases in low incidence geographical areas: involvement of numerous contagious and susceptible individuals, as well as rapid transfer of patients through modern transportation means, might create the epidemiological conditions for an unforeseen outbreak. Infectious diseases, rare in some geographic areas, can occur and rapidly spread in the context of unprepared national healthcare systems.

In addition, the number of international travelers is increasing globally and will be presumptively 1800 million by 2030 [1]. In total, 1326 million and 1401 million international travelers were recorded in 2017 and 2018, respectively. Between 2007 and 2011, infectious diseases were diagnosed in 42,173 travelers coming from Asia (32.6%), Sub-Saharan Africa (26.7%), and Latin America and the Caribbean (19.2%). Some of the infectious diseases were systemic (e.g., malaria, dengue, and enteric fever) and about one-third were caused by gastro-intestinal pathogens (e.g., *Campylobacter* spp., *Salmonella* spp., and *Shigella* spp.) [2,3]. Whereas a decreasing trend of incident malaria has been recorded from 2000 to 2015, incidence of enteric and dengue fever has not changed overtime [4].

Enteric fever, which includes Typhoid and Paratyphoid fever, is an infectious disease caused by *Salmonella enterica* serotypes *Typhi* and *Paratyphi* A, B and C. Their foodborne transmission, frequently associated with poor hygiene conditions and inadequate sanitation, favors outbreaks in low income countries [5]. Based on the most recent global estimates, ≥21 million incident cases and 222,000 typhoid-related deaths occur annually [6]. Improved sanitation and living conditions, as well as treatment of drinking water, have significantly contributed to decrease the incidence of enteric fever in high income countries (e.g., those located in Western Europe and North America). The Indian subcontinent and Southeast Asia show the highest annual incidence of typhoid fever (>100 cases per 100,000 cases annually), followed by Southern Africa (10–100 per 100,000 cases annually) [7,8]. In a recent meta-analysis conducted by Marchello and Colleagues [9], Africa and Asia were identified as high-endemic countries for typhoid fever, although a decreased trend in incidence was documented after 2000. Moreover, in low-resource areas, such as Tanzania, Myanmar, and Republic Democratic of Congo (DRC), *S. typhi* represents the leading cause of bloodstream infections in young children. In particular,>70% of cases occurred in children <10 years old and ~30% in <5 years old in DRC during 2015–2017. However, in high income countries, typhoid fever is one of the most frequently diagnosed vaccine-preventable diseases in returned international travelers and migrants coming from high incidence countries [10,11].

It has been estimated that the incidence rate of typhoid fever in travelers to high-endemic countries is 3–30 cases per 100,000 travelers [12].

A retrospective study carried out in the Netherlands from 1997 to 2014 found that the majority (59.6%) of patients with imported typhoid fever traveled in Asia (e.g., Indonesia (19.8%) and India (19.6%)), and Morocco (13.3%). A declining annual attack rate (i.e., annual incidence of imported cases to number of travelers in a geographical area) for all geographical destinations, with the only exception of India, has been described [13].

The more frequently affected age group was 25–29 years according to the findings of a survey performed in Australia, which confirmed East and South Asia as the highest risk geographical areas for individuals visiting their country of birth [14].Similar findings were confirmed by a Greek study which highlighted the risk of traveling in the Indian subcontinent during 2004–2011 (83.3% of the cases of travel-associated enteric fever), especially in VFR (Visiting Friends and Relatives)-travelers, whose disease is associated with longer stay, exposure to contaminated water and food, and difficult access to pre-travel medical services due to language and cultural barriers, as well as to lower rates of vaccination against travel-related preventable infections, including typhoid vaccine [15,16]. Similarly, a retrospective study conducted in Qatar, between 2005 and 2012, reported 356 cases of typhoid fever, of whom 96.9% had traveled abroad, mainly in the Indian subcontinent [17]. Over 70% of typhoid fever cases in Europe are acquired abroad and frequently caused by strains with marked antibiotic resistance profile [18,19].

In Italy, where typhoid fever was endemic in the first half of the last century, the mean annual notification rate was 127.6 cases during2007–2016. Although all cases were successfully treated, an unequal distribution of incident cases in the population group aged 25–44 years was found, likely linked to their travel habits [20].

When traveling from high- to low- and middle-income countries, the risk of infectious diseases is higher in VFR-travelers, followed by travelers for other reasons. Migrants from low income countries represent a vulnerable population group at highest risk of respiratory, vector- and food-borne diseases owing to the higher circulation of microorganisms in their country of origin. Moreover, the higher risk could depend on long periods of stay in the country of origin, often in remote rural areas where the healthcare infrastructures are poor, and on close contact with the local population, as well as on consumption of local food and water [21]. Frequent travels from/to high incidence countries increase the probability of acquiring infections, such as those caused by *Mycobacterium tuberculosis*, HIV, *Plasmodium* spp., and *Salmonella* spp. Ten years of surveillance in the UK demonstrated lower rates of enteric fever in UK-born vs. migrant populations. Migrants from South Asian countries are at highest risk of enteric fever (80% of the migrant cases) [22].

Another group at highest risk includes persons involved in humanitarian staffing (e.g., missionaries, medical, and humanitarian workers): their length of stay is long and their travel destinations are low income countries where the incidence of the above-mentioned infectious diseases is high. Nevertheless, the Global TravEpi Network (GTEN) data in US showed an appropriate pre-travel care and vaccination, over 90% of coverage, for hepatitis A, typhoid, and yellow fever [23].

Although epidemiological data revealed that the occurrence of typhoid fever cases in high-income countries is a rare event and the awareness, as well as the knowledge, of the disease is poor, up-to-date estimates of typhoid fever incidence could be useful in supporting prevention and vaccination national strategies. Moreover, it should be helpful to identify groups at high risk of infection to plan adequate preventive strategies. In addition, based on the poor specificity of typhoid fever symptoms, the potential diagnostic delay could increase the risk of a rapid spread in low-incidence areas.

## 2. Methods

A non-systematic, narrative review to retrieve the scientific evidence on imported enteric (i.e., typhoid and paratyphoid) fever diagnosed in high income countries was carried out to describe relevant clinical and public health features. The search engine PubMed was used to select peer-reviewed articles, published from 1January 2013 to 30 October 2019. References of the selected manuscripts were carefully assessed to detect important articles not included in the primary search. No detailed selection criteria were adopted to choose the articles. The following keywords were used to find articles on imported enteric fever-related diagnosis, therapy, epidemiology, and prevention: “typhoid fever”, “enteric fever”, and “travel”. In total, 207 records, published between January 2013 and October 2019, were found. Based on titles, abstracts, and full-texts, 71 (34.3%) studies were deemed suitable. Twenty-three (32.4%) were focused on epidemiological characteristics of enteric fever, fourteen (19.7%) on vaccines, fourteen (19.7%) on antimicrobial resistance of *Salmonella* spp. serotypes, ten (14.1%) on population groups at higher risk of acquiring typhoid fever, and ten (14.1%) on diagnosis and treatment.

### 2.1. Population at Higher Risk of Acquiring Typhoid Fever

The risk of typhoid fever and non-typhoidal Salmonella invasive infections is highest in infants, young children, and young adults with underlying comorbidities, including severe anemia, malaria, malnutrition, and HIV infection [24]. Moreover, recent reports from the international travelers agency showed that immunocompromised travelers, who usually follow the same itineraries of immunocompetent persons, visit countries at high risk of infections but the risk of developing travel-related diseases is five times higher if compared with that of immunocompetent persons [25].

However, data on groups at risk of acquiring typhoid infections are controversial and scant. Gordon showed that the immunological status cannot be associated with an increased risk or poor outcome. However, invasive diseases caused by non-typhoidal salmonellae are more frequently diagnosed in immunocompromised persons (e.g., persons with HIV/AIDS) [26]. Likewise, a study conducted in Africa did not find differences in HIV-positive patients and controls in the clinical presentation and outcomes of typhoid fever cases [27]. In contrast, Gotuzzo and Colleagues [28] found a rate of typhoid fever 25 times higher in HIV-positive patients than in the general population.

With the remarkable increased number of travelers from high-income countries during the last two decades, it was estimated that 1.9 million children traveled overseas every year from the United States; similarly, a significant increase of travelers (1.7 fold) was shown in Greece from 2004 to 2008 [29]. A high proportion of enteric fever cases was described in children aged 0–14 years (>26% in 2018) [30], mainly attributed to tourism and VFR-travels. Zhou and Colleagues highlighted an increased rate of childhood enteric fever in a large tertiary care center in Canada during 1985–2013, with several cases caused by *Salmonella paratyphi* A and B and by bacterial strains resistant to first-line antibiotics [31]. In Australia, 87% of the childhood cases were acquired mainly in Southeast Asia, with an annual increasing incidence from the period 2001–2005 (13 cases per year) to the period 2011–2015 (38 cases per year) [32]. Similar data were described in France, where children aged <18 years accounted for one-third of enteric fever patients, with 61% of the infections acquired in Africa [33].

Pre-travel counseling focused on hygiene and preventive measures could help reduce the risk of infection in individuals younger than two years, who cannot be immunized with the currently available vaccines [29].

### 2.2. Vaccination Status in Travelers

Infections can be averted with vaccines and hygiene-related recommendations. However, adherence to pre-travel advice, including the vaccination, is poor.

Since 2008, the World Health Organization has advocated the control of typhoid fever based on vaccine-related strategies. Bill and Melinda Gates Foundation launched in 2017 a partnership called “Typhoid Fever Vaccine Acceleration Consortium (TyVAC)”, mainly focused on children living in high endemic areas, to increase the prescription of the typhoid conjugate vaccine in Africa and Asia [34].

Currently, three typhoid vaccines are available:

- Live attenuated oral vaccine Ty21a
- Purified Vi capsular polysaccharide injectable vaccine
- Purified Vi polysaccharide conjugated parenteral vaccine

The effectiveness of the current vaccination strategies in travelers depends on several variables, such as previous immunizations, type, and length of travel. Poor awareness on the high risk of foodborne diseases in low- and low-middle-income countries can increase the pool of individuals with a vaccine hesitancy.

Surveys conducted in EU/EEA countries showed that hepatitis A is the first vaccine administered to Swiss (53%) and Italian (63%), travelers, followed by tetanus–diphtheria in Swiss (45.6%) and typhoid-fever vaccine in Italian (44.6%) travelers. Moreover, travel destinations can increase the request of pre-travel care (e.g., India and Thailand chosen for pleasure or business) [35,36]. Furthermore, low vaccine uptake and inappropriate precautions adopted by VFR-travelers was associated with a highest incidence of foodborne diseases [36,37].

A Greek survey focused on the administration of typhoid fever vaccines showed that a high proportion (44.2%) of travelers to India accepted the prescription of a vaccine, in comparison with a lower percentage of individuals traveling to Africa (~31%) [37].

Ty21a live attenuated vaccine, developed from attenuated Ty2 strains of *S. typhi*, does not confer protection after a single dose; three doses administered in alternate days in persons living in endemic countries and four doses in travelers are usually recommended to elicit adapted mucosal immunity (IgA antibodies), whose duration lasts ~7 years in 60–70% of the vaccinated cases [38,39]. Half of typhoid fever cases could be prevented until three years after vaccination [40].

Purified Vi capsular polysaccharide vaccine, administered in one single dose, is associated with a high immunogenicity (80–95%) in adults and children older than two years; nevertheless, Anwar and Colleagues reported a preventive effect between one third and one half of the cases in the first two years after the vaccination and no clear benefits after three years [41]. Moreover, an acceptable immunogenicity and safety profile was shown when co-administered with yellow-fever and quadrivalent meningococcal vaccines [42], as well as in children and HIV-positive individuals [38]. A second dose is recommended after three years [39].

Purified Vi polysaccharide vaccine can be conjugated to toxoids (Vi-diphtheria, Vi-Tetanus, and Vi-recombinant diphtheria CRM_197_) to increase IgG levels [38]. Two-year post-vaccination effectiveness was87% [36,43]. A single randomized trial in Indian children aged between six months and 12 years did not clarify the efficacy of two doses of Vi-TT vaccine one year after administration [40].A real-life survey in Germany described an adverse event rate of 28.6% (fatigue, pain, headache, pyrexia, myalgia, and swelling), increased after concomitant immunization with other vaccines(i.e., rabies, typhoid, and yellow fever vaccines) [44].A US military study showed more adverse events in individuals exposed to the polysaccharide Vi vaccine in comparison with those exposed to the oral vaccine, although rash and diarrhea were more incident in the latter group [45].Fever and pain at the injection site were incident using parenteral vaccines [40].

Several studies suggested a cross-protection against *S. paratyphi* A, B and C with the administration of the oral vaccine Ty21a (common O- and Vi-antigens) [38], even if a study which recruited US military personnel described a weak immunity against *S. paratyphi* A [46].

Primary prevention based on the available vaccines cannot be implemented in youngest children: the purified Vi capsular polysaccharide vaccine is not recommended in children aged <2 years for its poor immunogenicity; the live attenuated oral vaccine Ty21a is not well tolerated in children aged under five years [47].A recent review on the efficacy of the currently available vaccines confirmed that Ty21a and Vi polysaccharide vaccines can reduce the incidence of infections in adults and children aged >2 years [40].

### 2.3. Clinical Diagnosis and Treatment of Typhoid Fever

The clinical management of typhoid fever in travelers has significantly changed during the last two decades following the widespread distribution of MDR bacteria and the increased number of international travels in endemic countries, including high-risk individuals (e.g., children, immunocompromised patients, pregnant women, and elderly people) [48]. Although illness in travelers is usually not severe and self-limiting, urgent therapy can be needed to avoid life-threatening complications; then, a rapid diagnosis and therapy in returning travelers is key to avoid fatal consequences [49]. The information on the type of travel, including the collection of details such as accommodation and activities, as well as pre-travel immunization, could help in the differential diagnosis (e.g., malaria and dengue). The most prevalent symptoms in the case of typhoid infections are fever, diarrhea, vomiting, abdominal pain, and headache [31,50]. However, the specificity is poor and they can be attributed to other viral or bacterial agents, in both children and adults. Moreover, higher level of C-reactive protein can be also found in case of dengue and malaria. Typhoid fever is commonly characterized by gastrointestinal disorders [51,52,53] and, moreover, bradycardia (88%), and eosinopenia (63%) [54]. Empirical therapy is needed in the case of severe symptoms and when a rapid diagnosis cannot be performed.

As recommended by the international guidelines [24], a definitive diagnosis of typhoid fever requires cultural or molecular methods, with specimens collected ideally before the administration of an antimicrobial therapy. Blood is the preferred specimen. Although serologic tests are frequently requested, several studies highlighted their poor specificity and sensitivity. Antimicrobial susceptibility testing is strongly recommended for clinical (prescription of a tailored antibiotic therapy) and public health (surveillance) purposes.

Epidemiological investigations are key for the identification of the source case and of the contagious patients. Serotyping carried out by slide agglutination method and the characterization of the genomic profile using Pulsed Field Gel Electrophoresis (PFGE) and Multi Locus Sequence Typing (MLST) are recommended to confirm epidemiological links during a suspected outbreak.

In the era of multidrug resistant strains, the adoption of antimicrobial susceptibility testing is necessary to guide the choice of the most appropriate therapy, improving the clinical management, preventing relapse and the chronic carrier status. Europeans Committee on Antimicrobial Susceptibility Testing (EUCAST) and Clinical and Laboratory Standards Institute (CLSI) recommend the use of the following antibiotics to be tested in vitro for infections caused by *S. typhi* and *S. paratyphi*: ampicillin, chloramphenicol, cotrimoxazole, ciprofloxacin, ceftriaxone, and azithromycin (Table 1).

Patients are usually treated with oral antibiotics, antipyretic, and supportive therapy. Parenteral antibiotics are prescribed in complicated cases or when gastro-intestinal symptoms are persistent. Ciprofloxacin (15 mg/kg) for 5–7 days is prescribed in the case of moderate symptoms caused by fully susceptible isolates [55]. With the emergence of multidrug resistant strains, the first line treatment was represented by third generation cephalosporins, such as cefixime (20 mg/kg, max 200 mg) for 10–14 days. Alternative drugs are chloramphenicol (50–75 mg/kg/day) for 14–21 days, trimethoprim-sulfamethoxazole (8 mg/kg/day) for 14 days, or amoxicillin (75–100 mg/kg/day) for 14 days [56]. Cases of severe enteric fever, characterized by delirium, stupor, coma, or obtundation, can be successfully treated with corticosteroids (e.g., intravenous dexamethasone at an initial dose of 3 mg/kg, followed by 1 mg/kg every six hours for two days) [57].

### 2.4. Antimicrobial Resistance and Typhoid Fever

In the case of typhoid fever, the World Health Organization recommends notification to national authorities and drug susceptibility testing.

Until multidrug resistant strains emerged and spread in the second half of the 1980s, chloramphenicol, ampicillin, and co-trimoxazole were considered the first-line therapy of typhoid fever globally [58]. The indiscriminate use of antibiotics, particularly in low-income areas, has favored the selection and spread of antimicrobial resistant strains [59].

The first outbreak caused by chloramphenicol-resistant *S. typhi* occurred in Mexico in 1972, followed by notifications in other endemic areas (i.e., South Asia andAfrica) [60]. Moreover, ~66% of isolates in European countries [17], shows decreased susceptibility to ciprofloxacin, although it was considered in the past as first therapeutic choice [61] by the World Health Organization guidelines. In U.S., during 2016–2018, 5/29 cases of typhoid fever in patients who recently traveled to Pakistan were caused by extensively drug resistant strains [18].

Data of the US antimicrobial monitoring system showed that >60% of the isolates had a reduced susceptibility to ciprofloxacin and nalidixic acid, as well as alarming increasing trends of resistance of *S. typhi* and *paratyphi* A [62] (Table 2).

Britto and Colleagues systematically reviewed historical trends of drug resistance to first-line antimicrobial therapy in typhoid fever (1973–2018, for 13,833 isolates) (Table 3).

The majority were isolated in South Asia (63.2%), Africa (15%), and Southeast Asia (12.8%). Moreover, during 2006–2015, a significant increasing trend of drug resistance was described for the nalidixic acid and fluoroquinolones [63].

The increasing rate of *S. typhi* isolates resistant to quinolones, ampicillin, and cotrimoxazole prompted a policy change in terms of antibiotic prescription, mainly focused on third-generation cephalosporines [31,32]. Strains of multi-drug resistant (MDR) *Salmonella typhi* with the haplotype H58 were found worldwide, and accounted for multiple outbreaks in endemic countries and single cases in international travelers. Moreover, Klemm and Colleagues [64] detected several resistance genes (extended-spectrum *β*-lactamase genes, and ESBLs) in extensively-drug resistant strains, previously detected in other enteric bacteria [65]. Resistance is mainly associated with cumulative mutations in quinolone resistance-determining regions (QRDR) encoding DNA gyrase (*gyrA* and *gyrB* genes) and topoisomerase (*parC* and *parE* genes) [61,66]. A recent study, which analyzed >500 isolates of *S. typhi*, found that the reduced susceptibility to fluoroquinolones is frequently (85%) associated with point mutations in the QRDR; however, the prevalence can vary depending on the geographical area:95% in South Asia, 43% in EastAfrica, and 27% in WestAfrica. Strains resistant to ciprofloxacin were mainly collected from patients diagnosed in India, accounting for 23% of the total Indian cases [67].

*Qnr*-family genes, which are responsible of plasmid-mediated quinolone resistance, confer resistance to nalidixic acid and are recommended as a surrogate marker of decreased susceptibility to ciprofloxacin [66].

Recent reports from high-income countries described trends of decreased susceptibility for several antimicrobial drugs [61,66,68]: ciprofloxacin-resistant strains were 63%, 55.6%, and ~43% in Italy, Switzerland, and Spain, respectively. Antimicrobial resistance is frequently reported in USA where 69% of strains isolated between 2008 and 2012 were resistant to nalidixic acid or showed reduced susceptibility to ciprofloxacin, 12% were MDR, and 10% showed extensive drug resistance [69]. Furthermore, the poor antimicrobial susceptibility was found in both *S. parathypi* and *S. typhi* isolates; drug resistance to quinolones was detected in 66.7% and 20% isolates of *S. paratyphi* and *S. typhi*, respectively [68].

Third generation cephalosporins (e.g., ceftriaxone) are successfully prescribed for infections caused by ciprofloxacin-resistant strains; alternatively, meropenem and aztreonam can be administered, even if the clinical breakpoint for aztreonam is not currently available [70]. Furthermore, 354 isolates of *S. typhi* collected in the Netherlands showed resistance to ciprofloxacin and azithromycin, reducing the availability of effective therapeutic options, mainly in travelers with acquired infections in endemic settings [71].

On this basis, World Health Organization recommends the management of sporadic cases of typhoid fever diagnosed in high-income countries be performed in reference centers, where a comprehensive assessment, including the complete drug susceptibility testing, could be carried out [24].

## 3. Conclusions

The present narrative review highlights that typhoid fever is an important clinical and public health issue in returning travelers. More international efforts and cooperation activities should be planned to reduce the burden of foodborne diseases, including typhoid fever, in low- and middle-income countries, addressing the key role played by the global surveillance systems. In particular, reliable and real-time estimates of typhoid fever can help better assess the efficacy and cost-effectiveness of ad hoc activities realized in both low and high endemic countries. The knowledge of the healthcare workers on travel related-diseases should be improved, focusing on the importance of a rapid diagnosis, including a rapid drug susceptibility testing, for the identification of difficult-to-treat cases (e.g., patients with typhoid fever caused by multi-drug resistant strains).

Primary prevention strategies and pre-travel health advices could decrease the risk of acquiring infections: improved awareness in community members could support and improve the adherence, mainly in highly susceptible groups (e.g., children and immunocompromised patients).

Nevertheless, further epidemiological studies should be focused on the effectiveness of currently available vaccines in population groups poorly evaluated in studies and trials carried out in the past. International surveillance networks involving endemic countries are needed to rapidly detect the emergence and spread of drug-resistant bacteria and decrease the incidence of imported cases in high-income countries. The financial and economic impact of endemic diseases can be easily reduced improving hygiene conditions in constrained-resource countries; however, only the improvement of political and economic issues can be achieved with comprehensive interventions supported by the international community, which directly and indirectly can affect the epidemiology of life-threatening diseases.

## Figures and Tables

**Table 1:** Clinical breakpoints recommended by EUCAST and CLSI. (NA, Not Applicable).

Antibiotic	CLSI MIC Interpretative Criteria (g/mL)			CLSI Zone Diameter Interpretative Criteria (Nearestwhole mm)				EUCAST MIC (mg/L)		EUCAST Zone Diameter Breakpoint (mm)		
	S	I	R	Disk Content µg	S	I	R	S	R	Disk Content µg	S	R
Ampicillin	≤8	16	≥32	10	≥17	14–16	≤13	≤8	≥8	10	≥14	≤14
Cefotaxime	≤1	2	≥4	30	≥26	23–25	≤22	≤1	≥2	5	≥20	≤17
Ciprofloxacin	≤0.06	0.12–0.5	≥1	5	≥31	21–30	≤20	≤0.06	≥0.06	5	≥24	≤24
Levofloxacin	≤0.12	0.25–1	≥2	-	-	-	≤0.12	≤1	≥2	5	≥22	≤19
Oflaxacin	≤0.12	0.25–1	≥2	-	-	-	≤0.12	≤0.1	≥1	5	≥22	≤19
Nalidixic acid	≤16	-	≥32	30	≥19	14–18	≤13	NA	NA	-	NA	NA
Trimethoprim/sulfamethoxazole	≤2/38	-	≥4/76	1.25/23.75	≥16	11–15	≤10	≤2	≥4	1.25/23.75	≥16	≤13
Chloramphenicol	≤8	16	≥32	30	≥18	13–17	≤12	≤8	≥8	30	≥17	≤17

**Table 2:** Antimicrobial resistance pattern for *Salmonella* ser. *Typhi* (No. of isolates = 336), *Paratyphi* A (No. of isolates = 88), 2015 (CDC-National Antimicrobial Resistance Monitoring System-2015).

Antibiotics	Percentage Susceptible		Percentage Intermediate		Percentage Resistant	
	*S. typhi*	*S. paratyphi A*	*S. typhi*	*S. paratyphi A*	*S. typhi*	*S. paratyphi A*
*Gentamicin*	100	100	0	0	0	0
*Streptomycin*	84.5	90.9	N/A	N/A	15.5	9.1
*Amoxicillin-clavulanic acid*	98.8	100	1.2	0	0	0
*Ceftiofur*	100	100	0	0	0	0
*Ceftriaxone*	100	100	0	0	0	0
*Azithromycin*	99.7	100	N/A	N/A	0.3	0
*Ampicillin*	89.6	98.9	0	0	10.4	1.1
*Ciprofloxacin*	34.3	11.4	57.4	88.6	8.3	0
*Nalidixic acid*	36.6	11.4	N/A	N/A	63.4	88.6
*Cefoxitin*	99.4	96.6	0.6	2.3	0	1.1
*Sulfisoxazole*	88.4	97.7	N/A	N/A	11.6	2.3
*Trimethoprim-sulfamethoxazole*	88.1	98.9	N/A	N/A	11.9	1.1
*Chloramphenicol*	90.5	94.3	0	5.7	9.5	0
*Tetracycline*	96.1	94.4	1.2	4.5	2.7	1.1

**Table 3:** Proportion (%) of drug-resistant isolates of *Salmonella typhi*.

Drug	Proportion (%) of Drug-Resistant Isolates					
	Pre 1991	1991–1995	1996–2000	2001–2005	2006–2010	2011–2015
Cholamphenicol	31.2	49.2	44	31	19	13
Ampicillin	16.2	49.1	46	35	32	20
Cotrimoxazole	16.1	49.2	45	33	18	18
Nalidixic Acid	0	0	22	50	63	80
Ciprofloxacin	0	0	12	23	33	63
Cephalosporins	0	0	2	1	1	4
